# State of Accredited Endovascular Neurosurgery Training in India in 2021: Challenges to Capacity Building in Subspecialty Neurosurgical Care

**DOI:** 10.3389/fsurg.2021.705246

**Published:** 2021-08-31

**Authors:** Saisunder Shashank Chaganty, Ahmad Ozair, Faique Rahman

**Affiliations:** ^1^University Hospitals North Midlands, Stoke-on-Trent, United Kingdom; ^2^Faculty of Medicine, King George's Medical University, Lucknow, India; ^3^Jawaharlal Nehru Medical College and Hospital, Aligarh Muslim University, Aligarh, India

**Keywords:** global health (MeSH [H02.403.371]), global surgery, medical education, postgraduate training, clinical training, neurointervention, neurology, stroke

## Introduction

Stroke is the second leading cause of death worldwide, accounting for nearly 5.5 million deaths annually along with significant long-term disability. Currently, there exist >80 million stroke survivors worldwide and the condition is responsible for 5.2% of disability-adjusted life years (DALYs) lost globally (1, 2). Stroke is not just a clinical condition but a massive public health emergency for which work is needed throughout the healthcare spectrum—from prevention to optimal management. While the former is primarily the domain of primary care and public health, the latter falls in the domain of specialist care. The burden of stroke, however, is disproportionately borne by developing nations, which house a large proportion of the world's population.

Endovascular neurosurgical care has been demonstrated to be a safe and highly efficacious treatment strategy for stroke and related conditions, such as aneurysms, subarachnoid hemorrhage, carotid artery disease, amongst others (3, 4). Neuroendovascular specialists in HICs are either interventional neuroradiologists, interventional neurologists, or neurosurgeons. Given that endovascular therapy has especially proved to be superior to intravenous thrombolysis in acute ischemic stroke (AIS) and can be utilized beyond 6 hours of symptom onset with appropriate patient selection, neurointerventional management has hence become a standard of care in high-income countries (HICs) (3).

Unfortunately, the above situation is almost exclusive to HICs with endovascular care being neither available nor widely accessible in low-middle-income countries (LMICs). Several issues have come together to result in a state of significant disparity in the availability of endovascular care, ranging from infrastructural cost considerations to the presence and distribution of trained specialists. However, it is in LMICs where the greatest need for endovascular neurosurgery exists. For instance, stroke in South Asia, relative to HICs, is known to have a higher prevalence, younger age, higher mortality, and an increased burden of modifiable risk factors (5). In addition, neuroendovascular specialists in LMICs are predominantly neuroradiologists, given that radiologists were the earliest in large parts of the world to venture into this subspeciality.

The primary specialty of the neurointerventionalist has been demonstrated to be of significant value. In a seminal paper in 2016, Fennel et al. reported that, for endovascularly treated intracerebral aneurysms, neurologists had the highest rates of complications (11%) and deaths (3%), followed by radiologists, while neurosurgeons had the lowest rates of complications (5.4%) and mortality (1.4%) (6). These large differences suggest that endovascularly-trained neurosurgeons should be one of the key neuroendovascular subspecialists available worldwide.

India, in particular, has seen a rise in stroke cases from 2.8 million in 1990 to 6.5 million in 2016 (2). However, the state of endovascular care services by neurosurgeons in India remains nearly non-existent, as we describe below. This opinion piece utilizes the Indian case scenario to highlight how the disparity of endovascular neurosurgical care exists against excellent training being available for general neurosurgery and its other subspecialties (7). The challenges in India echo those of other LMICs with regards to capacity building in high-quality neurosurgical care in various subspecialties.

## Neurosurgical Residency Training in India

To put the near absolute lack of formally trained endovascular neurosurgeons in India in context, one must first understand the state of neurosurgical residency training in India, which is currently quite well-developed in number and distribution (7).

After 5.5 years of medical school, graduates then sit for annually held, national, standardized examinations. Ranks on these examinations, now consolidated into just two, serve as the sole basis for selection into all residency programs and all specialties. Two types of residency training programs exist, those accredited by the National Board of Examination (NBE) and those accredited by the National Medical Council (NMC), erstwhile known as the Medical Council of India (MCI). For all specialties, the NMC-accredited programs are usually at publicly-funded, non-profit institutions, while the NBE-accredited programs exist typically at private hospitals and/or for-profit institutions. Completion of neurosurgery training leads to the award of Magister Chirurgiae (M.Ch.) if completed at NMC-accredited institutions or the award of Doctorate of the National Board in Super-Speciality (DNB-SS) if completed at NBE-accredited residencies.

**Figure 1:**
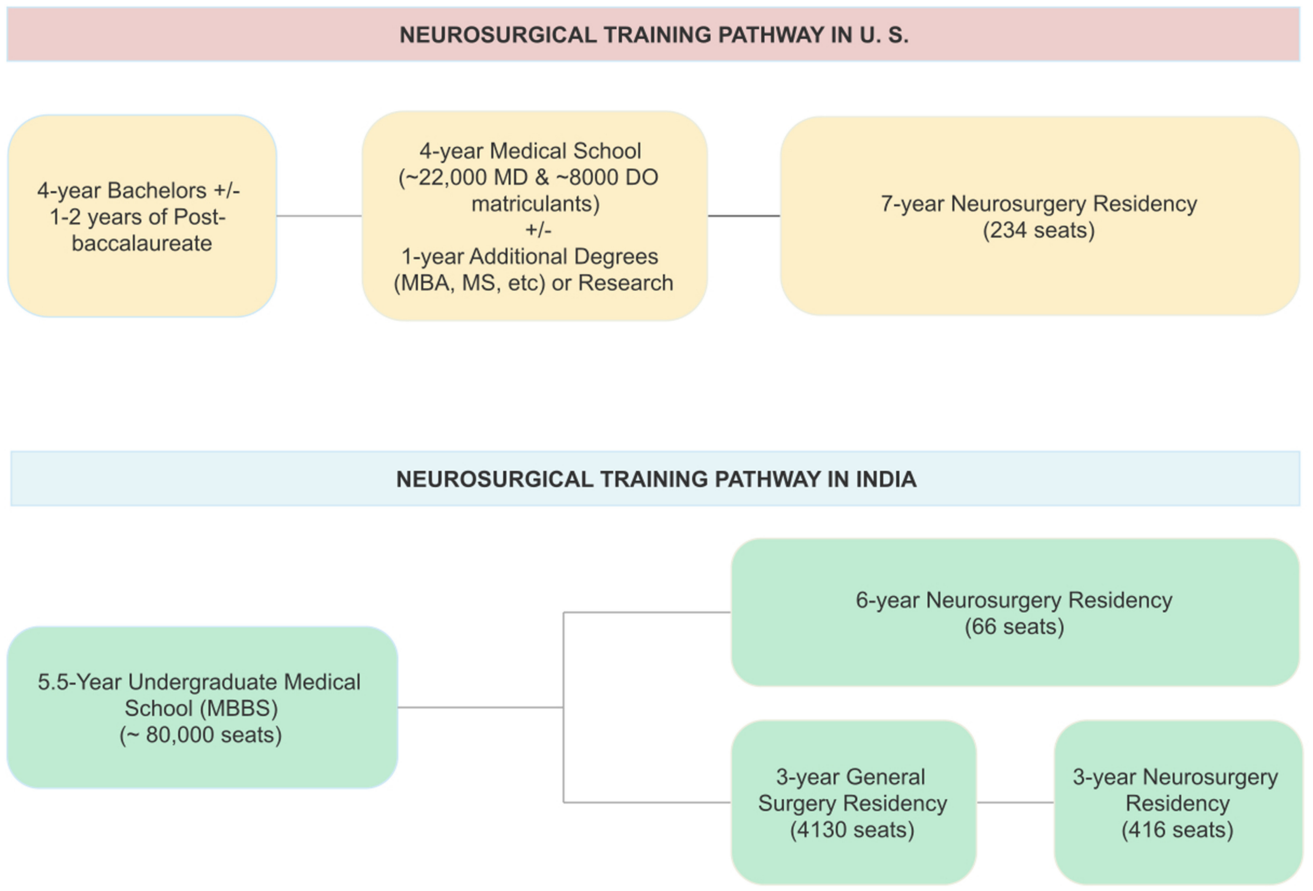
Comparison between the United States and India of training lengths and positions for the training of a neurosurgeon. Seat numbers in India are sum totals for programs accredited by either National Medical Council (NMC) or National Board of Examinations (NBE) in India.

Neurosurgery training in India is currently through two pathways (Figure 1). The first pathway comprises of 3-year-long programs, whose selection test is given after completion of a 3-year general surgery residency. This pathway provides the bulk of neurosurgical training positions in India. The other, which is far more limited, includes 5/6-year-long programs straight after medical school. The latter is quite similar to all neurosurgery training positions in the US, Canada, and to the run-through programs in the United Kingdom (Figure 1).

The number of neurosurgery, neurology, and neuroradiology training programs in India is given in Supplementary Table 1 (8, 9). India, as of May 2021, has a total of 482 accredited intake positions for neurosurgery training annually, likely the highest number worldwide. Notably, neurology training in India is solely a 3-year program, done after a 3-year training in internal medicine. It leads to the award of the degree of Doctorate of Medicine (DM) at MCI/NMC-accredited institutions or the DNB-SS by the NBE-accredited residencies. This is in contrast to the US where neurology is a 4-year residency program after medical school. With regards to neuroradiology, it is a 3-year program after 3-years of radiology residency. Most neuroradiology programs in India have some degree of training in neurointerventional management.

## State of Subspecialty Training in Endovascular Neurosurgery in India

Currently, for endovascular training for neurosurgery residency neurosurgery, there exist zero training programs in India accredited by the MCI/NMC. For the same, a 2-year program accredited by NBE was introduced in 2020, open not just to neurosurgeons but to radiologists and neurologists as well, and awards the degree of Fellow of the National Board (FNB) in Neurovascular Intervention. It currently has just 4 training positions at a total of 3 programs (Supplementary Table 1). With regards to non-accredited training, there exist a handful of centers offering endovascular neurosurgery training, but their number is extremely limited, the duration is highly variable and the training is non-standardized (10, 11). Thus, despite the adequate state of neurosurgery residency training, there simply exists little high-quality training to become an endovascular neurosurgeon in India.

Currently, there exist 7 NMC-accredited neuroradiology programs offering 22 seats annually. Notably, all of them offer some degree of neuro-interventional training. However, these are open to apply only to radiologists while also having a large degree of heterogeneity in trainee procedural volume.

In contrast, other neurosurgical subspecialties have well-developed training programs in India. While several reasons for this disparity exist, one major factor is the need for the additional logistical framework for delivering endovascular care, like dedicated catheterization labs. This is in contrast to other neurosurgical subspecialties that primarily utilize the pre-existing infrastructure such as complex skull-base surgery.

## Discussion

Understanding the current accessibility of endovascular neurosurgery training in India is thus an essential first step to address the growing disparity in service provision and to develop recommendations to improve training systems (12–14).

In the US, there are ~116 neuroendovascular fellowship programs for neurosurgeons that are accredited and/or listed by the Society of Neuro-interventional Surgery (SNIS), Committee on Advanced Subspecialty Training (CAST), and Accreditation Council for Graduate Medical Education (ACGME) (15–17). Another accrediting body for neurointerventional fellowships is United Council for Neurologic Subspecialties (UCNS). Notably, it is the ACGME that accredits all residency training in US. In India, equivalent nationally recognized bodies exist such as NMC and NBE, along with professional societies like the Neurological Society of India (NSI) and the Neurological Surgeons Society of India (NSSI). However, accrediting such fellowships is not a part of their scope to date.

In a recent national survey study of 104 Indian neurosurgical trainees, 61% described exposure to endovascular surgery as uniformly poor (18). In the coexistence of both poor exposure during residency and complete dearth of experience via accredited fellowships, it is likely that those neurosurgical trainees initially interested in neuroendovascular turn to other subspecialties.

Amidst such sentiments, it is not surprising that many young Indian neurosurgeons have to apply for and complete training in developing nations to become competent in endovascular neurosurgery. This carries its own challenges, as countries differ with regard to how they define fellowship. It has been noted by prominent academic neurosurgeons in India that some neurosurgeons undertake only a few weeks of observership abroad and return to India declaring themselves as endovascular neurosurgeons (12, 19). Some who complete fellowships in North America choose to practice there.

Thus, with endovascular neurosurgery enthusiasts either turning to other specialties or seeking opportunities abroad, the specialty's workforce growth stagnates, resulting in less momentum for the development of training programs, and hence a vicious circle perpetuates. Therefore, the first and the most important point of input must be into starting accredited programs for endovascular training at major neurosurgical departments in the country.

Secondly, these national bodies, working with local non-profit organizations, must commission and/or fund epidemiological studies looking to conclusively quantify the need for endovascular neurosurgeons. This data may then be utilized to request greater funding from federal agencies for such subspecialists. Given India's demographic transition from a developing into a developed nation, which carries with itself a greater prevalence of obesity, hypertension, dyslipidemia, and thereby stroke, the need has only grown in recent decades.

Thirdly, given the non-accredited nature of the vast majority of neurosurgery subspecialty fellowships in India (7, 10, 11), professional neurosurgical societies must play a key role in either beginning fellowship accreditation themselves or supporting external accrediting agencies. This may be done by developing strict procedural volumes, training outcomes, and milestones-based competency criteria, which may be adapted from the standards adopted by ACGME and CAST based on the case-mix and disease burden in India. Notably, lack of fellowship accreditation in other neurosurgical subspecialties has led to significant heterogeneity in their training quality and the operative volume, a scenario which can be preemptively avoided in the case of endovascular fellowships given its blank slate in India.

Additionally, the Indian neurosurgical professional bodies would do well to increase collaboration with international organizations to build capacity. The NSI is a particular leader that could forge directed collaborations for developing the training of endovascular neurosurgeons with organizations such as the World Federation of Neurosurgical Societies (WFNS), the American Association of Neurological Surgeons (AANS), Congress of Neurological Surgeons (CNS), and the European Association of Neurosurgical Societies (EANS) (14). Such mechanisms would allow for a large expert collaborative community which is essential for the exposure required by the early-career neurosurgeons interested in endovascular neurosurgery. The collaboration would allow high-quality training programs in India to develop having been mentored by global experts from the start.

Furthermore, given that most neurosurgery training programs in India lack neuroendovascular rotations for residents, a major reform would be the incorporation of such rotations during residency, similar to programs globally. Six-year training programs are especially poised to offer a 1-year enfolded training option, including in endovascular neurosurgery, similar to the enfolded fellowship model in the US.

Finally, in conjunction with previous literature, we recommend expanding the number of 6-year programs and converting the 3-year pathways to 6-year ones, given that 3 years has been noted to be too little a time to train competent and safe neurosurgeons (7, 10, 11). This will greatly help training programs in accommodating a neurointerventional rotation for trainee neurosurgeons. Herein, thought must also be given to providing trainees early autonomy and decreasing the amount of scut work, two considerations that have long plagued neurosurgical training in India (7, 10, 11, 18).

In summary, these recommendations represent the first steps toward raising the profile and the value of endovascular neurosurgery amidst trainees and the wider neurological care systems in India (Supplementary Figure 1). Despite this, it is important to acknowledge other considerations that would impede its growth including expanding rural-urban divides, health commodification, misgovernance, and bureaucratic apathy (20). For a call for more endovascular neurosurgeons is a recommendation laced with an assumption that the Indian ecosystem would support this; based on these external factors it would be like building the second floor before laying the brick and cement. It is only by addressing these systemic issues alongside the recommendations laid out herein that Indian neurosurgeons can strive toward a high-quality endovascular neurosurgical workforce in India.

## Author Contributions

SSC and AO conceptualized, drafted and revised the manuscript. FR drafted and revised the manuscript. All authors contributed to the article and approved the submitted version.

## Conflict of Interest

The authors declare that the research was conducted in the absence of any commercial or financial relationships that could be construed as a potential conflict of interest.

## Publisher's Note

All claims expressed in this article are solely those of the authors and do not necessarily represent those of their affiliated organizations, or those of the publisher, the editors and the reviewers. Any product that may be evaluated in this article, or claim that may be made by its manufacturer, is not guaranteed or endorsed by the publisher.
