# Supplementary material for: State of Accredited Endovascular Neurosurgery Training in India in 2021: Challenges to Capacity Building in Subspecialty Neurosurgical Care
Source: Front Surg. 2021 Aug 31;8:705246. doi: 10.3389/fsurg.2021.705246 (PMC8448280; doi:10.3389/fsurg.2021.705246)

SUPPLEMENTARY MATERIAL

State of Accredited Endovascular Neurosurgery Training in India in 2021: Challenges to Capacity Building in Subspecialty Neurosurgical Care

Authors: Saisunder Shashank Chaganty, Ahmad Ozair, Faique Rahman

doi: 10.3389/fsurg.2021.705246

Index:

Page 2: Supplementary Table 1

Page 3: Supplementary Figure 1

Supplementary Table 1: Current state of neurosurgery, neurology and neuroradiology training in India, with data current as of May 4, 2021, from the websites of the National Medical Council (NMC) and the National Board of Examination (NBE). Note that the NMC website classifies all ten seats of neurosurgery at National Institute of Mental Health & Neuro Sciences (NIMHANS) into the 3-year program, however that is the case only when no candidates in the 6-year program are found eligible. Typically 4 candidates are admitted in the 6-year course and 6 are admitted in the 3-year course at NIMHANS. The table has been modified to reflect the typical scenario.

Type of Training	Eligibility for Training	NMC-accredited		NBE-accredited	
		No. of programs	No. of seats	No. of programs	No. of seats
3-year Neurosurgery	Completion of 3-year general surgery residency	98	350	44	66
6-year Neurosurgery	Completion of medical school	3	21	22	45
3-year Neurology	Completion of 3-year internal medicine residency	89	337	74	150
3-year Neuro-Radiology & Neurointervention	Completion of 3-year radiology residency	7	25	-	-
2-year Neurovascular Intervention	Completion of neuro-radiology, neurology or neurosurgery training	-	-	3	4

Supplementary Figure 1: Recommendations for capacity building in endovascular neurosurgery training in India.

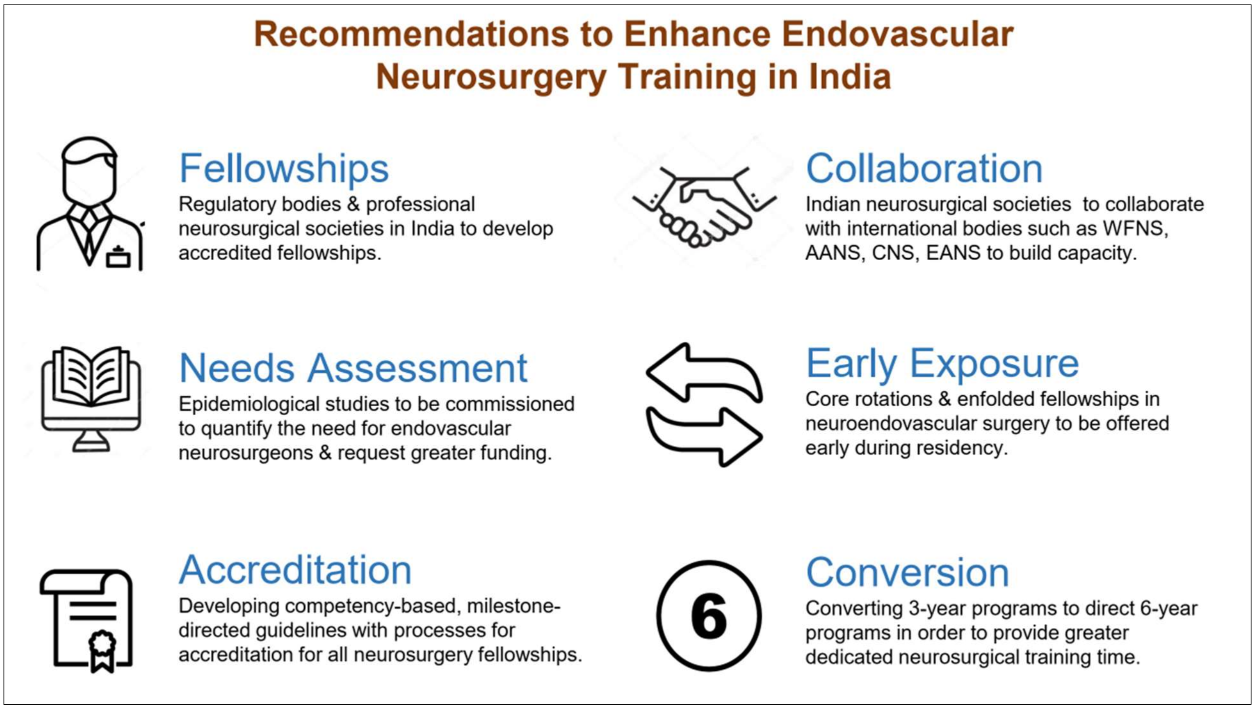

Supplement: Supplementary file 1 [file Data_Sheet_1.pdf]
